# The acquisition of the gender‐brilliance stereotype: Age trajectory, relation to parents' stereotypes, and intersections with race/ethnicity

**DOI:** 10.1111/cdev.13809

**Published:** 2022-05-30

**Authors:** Siqi Zhao, Peipei Setoh, Daniel Storage, Andrei Cimpian

**Affiliations:** ^1^ Psychology, School of Social Sciences Nanyang Technological University Singapore; ^2^ Department of Psychology University of Denver Denver Colorado USA; ^3^ Department of Psychology New York University New York New York USA

## Abstract

Past research has explored children's gender stereotypes about specific intellectual domains, such as mathematics and science, but less is known about the acquisition of domain‐general stereotypes about the intellectual abilities of women and men. During 2017 and 2018, the authors administered Implicit Association Tests to Chinese Singaporean adults and 8‐ to 12‐year‐olds (*N* = 731; 58% female) to examine the gender stereotype that portrays exceptional intellectual ability (e.g., genius, brilliance) as a male attribute. This *gender‐brilliance stereotype* was present among adults and children and for both Chinese and White stereotype targets. It also was stronger among older children and among children whose parents also showed it. This early‐emerging stereotype may be an obstacle to gender equity in many prestigious employment sectors.

**Abbreviations**
CI: confidence interval
IAT: Implicit Association Test
SC: single‐category
SGD: Singapore dollars
STEM: science, technology, engineering, and mathematics

Gender imbalances persist in science, technology, engineering, and mathematics (STEM) careers and are unlikely to disappear in the near future (United Nations Educational, Scientific and Cultural Organization, 2020; World Economic Forum, 2019). Along the school‐to‐career pathway, fewer women enroll in STEM programs, and even fewer women undertake professional roles in STEM fields (World Economic Forum, 2019). What causes these gender imbalances? One possible explanation is the presence and early acquisition of gender stereotypes (e.g., Boston & Cimpian, 2018; Cheryan et al., 2015). Such stereotypes may shape girls' and boys' interests and pursuits, steering them away from certain domains and toward others. Past research has explored the development and implications of children's gender stereotypes about specific intellectual domains, such as math and science (e.g., Cvencek et al., 2011, 2015; Galdi et al., 2014; Starr & Simpkins, 2021; Steffens et al., 2010). However, domain‐*general* stereotypes about the intellectual abilities of women and men also exist and perpetuate unfavorable views of girls and women (Bian, Leslie, & Cimpian, 2018; Bian et al., 2017; Jaxon, Lei, et al., 2019; Syzmanowicz & Furnham, 2011). Such a stereotype is our focus here: We examined the domain‐general gender stereotype that portrays exceptional intellectual ability as a male attribute (Bian et al., 2017; Storage et al., 2016, 2020). This *gender‐brilliance stereotype* may adversely affect women's prospects in STEM—among other domains—because these disciplines are often perceived as requiring a particularly high level of intellectual ability (Bian, Leslie, & Cimpian, 2018; Bian, Leslie, Murphy, et al., 2018; Ito & McPherson, 2018; Leslie, Cimpian, et al., 2015; Muradoglu et al., 2021).

In the present studies, we extend previous research on the gender‐brilliance stereotype by exploring (1) its age trajectory across the elementary school years, as well as (2) the relation between children's and their parents' gender‐brilliance stereotypes and (3) this stereotype's intersection with stereotypes about race/ethnicity. In addition, the present research contributes to the literature on the gender‐brilliance stereotype by going beyond the U.S. samples in which this stereotype has primarily been studied so far and investigating it in a meaningfully different cultural context: Singapore. In the following sections, we review prior work on the gender‐brilliance stereotype, highlighting the open questions that the present research will answer.

## The age trajectory of children's gender‐brilliance stereotype

The gender‐brilliance stereotype takes root surprisingly early. Rather than being endorsed only by adults (e.g., Storage et al., 2016), it seems to be present even as early as age 6 (Bian et al., 2017; Jaxon, Lei, et al., 2019). Bian et al. (2017) assessed this stereotype among a sample of 5‐ to 7‐year‐olds from the Midwestern United States. In this sample, 6‐ and 7‐year‐old girls (but not 5‐year‐old girls) were less likely to attribute brilliance to their own gender than were boys of the same age (Bian et al., 2017; see also Bian, Leslie, & Cimpian, 2018). Jaxon, Lei, et al. (2019) replicated this finding in a sample of 5‐ and 6‐year‐olds from the Northeastern United States. Finally, Storage et al. (2020) used an implicit measure of stereotyping (the Implicit Association Test [IAT]) and found the stereotypical “brilliance = men” association in samples of 9‐ to 10‐year‐old children from the Midwestern and Northeastern United States, as well as adults from the same regions.

A question that is left open by these prior studies concerns the developmental trajectory of the gender‐brilliance stereotype: Once this stereotype emerges, does it undergo change, or are its initial levels (i.e., among 6‐year‐olds) similar to what we observe in adults? This question is important from both a theoretical and a practical standpoint (e.g., the age trajectory of the stereotype can suggest potential sources), but it remains unanswered. Bian et al. (2017) observed no changes in the gender‐brilliance stereotype after age 6, but this study's ability to detect such age‐related changes was limited by the fact that it only included children up to the age of 7. The age range of Storage et al.'s (2020) sample was also limited (9‐ and 10‐year‐olds). Moreover, the fact that Storage et al. used a different (implicit) measure of stereotyping makes it difficult to compare their data with those of Bian et al. (2017) and Jaxon, Lei, et al. (2019).

Prior work on children's endorsement of gender stereotypes across childhood and adolescence licenses competing predictions about the trajectory of the gender‐brilliance stereotype after the age of 7. On the one hand, we might expect this stereotype to *increase* in strength with age. For instance, as children grow older, they accumulate exposure to cultural messages that associate brilliance with men (e.g., Avitzour et al., 2020; J. Cimpian et al., 2016). If children absorb these messages, then the extent to which they themselves associate brilliance with men should show a corresponding increase. On the other hand, there are also reasons to expect the gender‐brilliance stereotype might *decrease* with age. Older children tend to exhibit greater flexibility in their attitudes about gender (e.g., Alfieri et al., 1996; Kurtz‐Costes et al., 2008; Trautner et al., 2005), and their gender‐egalitarian tendencies also increase with age in some respects—for instance, older children are more likely to say that women and men pursue (and *should* pursue) the same types of activities and occupations (e.g., Liben & Bigler, 2002; Trautner et al., 2005; see also Halim et al., 2011). Over time, these developments may make it less likely that children view brilliance as a male quality. Of course, other developmental trajectories are possible as well. For example, the gender‐brilliance stereotype may also remain stable after it is acquired (e.g., if the two processes above counteract each other's effects) or exhibit a more complex, U‐shaped developmental trajectory (e.g., Raabe & Beelmann, 2011).

To get an initial sense of which age trajectory is more likely, we can look to previous research on gender stereotypes about intellectually relevant domains (e.g., math, science, reading/writing). However, the relevant results are mixed. On the one hand, some past studies have found age‐related increases in the magnitude of gender stereotypes about specific intellectual domains, with older children associating boys (vs. girls) more strongly with mathematical and scientific ability (e.g., Miller et al., 2018; Muzzatti & Agnoli, 2007) and girls (vs. boys) more strongly with verbal ability (e.g., Heyman & Legare, 2004; Rowley et al., 2007), perhaps in part because of continued exposure to their parents' and teachers' own stereotypes (e.g., Gunderson et al., 2012). In contrast, other studies found little change in the same stereotypes, even across a sizable age span (e.g., Cvencek et al., 2011, 2015; Heyman & Legare, 2004), and some even found decreases (e.g., Kurtz‐Costes et al., 2008). Thus, previous research does not license strong expectations about the age trajectory of the gender‐brilliance stereotype. Further evidence is needed to understand whether and how this stereotype changes with age.

To explore this developmental trajectory, in the present research we included the widest age range to date in research on this topic: 5 years (8‐ to 12‐year‐olds). The age of 8 was chosen as the lower limit of the age range in part because prior work suggested that changes in the strength of the gender‐brilliance stereotype are unlikely among 6‐ and 7‐year‐olds (Bian et al., 2017; Jaxon, Lei, et al., 2019). Notably, we also recruited a larger sample (*N* = 342) than in previous work to be able to detect even subtle shifts in the gender‐brilliance stereotype with age.

## The relation between children's and their parents' gender‐brilliance stereotypes

Little is known about the sources of the gender‐brilliance stereotype in children's environments: Where and/or who do children acquire this stereotype from? In the present research, we investigated the possibility that children's stereotypes on this topic are related to *their parents' stereotypes*. Parents act as gender socialization agents (e.g., Gunderson et al., 2012; Maccoby, 1992), shaping their children's gender beliefs through various means, such as expressing gender norms, fostering children's same‐gender and cross‐gender interests, and modeling gender‐role‐related behaviors (e.g., Crowley et al., 2001; Epstein & Ward, 2011; Kirkcaldy et al., 2007; Tenenbaum & Leaper, 2002; Tiedemann, 2000). Importantly, children seem to notice and internalize their parents' views of gender (e.g., Degner & Dalege, 2013; Endendijk et al., 2013; Starr & Simpkins, 2021; but see del Río et al., 2018; McHale et al., 1999). In a meta‐analysis, for instance, Degner and Dalege (2013) found that parents' and their children's gender‐related attitudes were positively correlated, with an overall effect size of *r* = .21. Additionally, the parent–child attitude association was present regardless of the parent's gender, and the strength of the association increased with children's age.

To investigate whether parents' and children's gender‐brilliance stereotypes are related, in the present research, we measured the extent to which parents and their children associate brilliance and genius with men more than women. To provide a sensitive test of this relationship, we used the same measure in both parents and children: namely, a gender‐brilliance IAT (Storage et al., 2020). The IAT is well suited for our purposes here because it is less susceptible to socially desirable responding than explicit measures of stereotyping (e.g., Greenwald et al., 2009). Thus, using the IAT allows us to bypass any reluctance on the part of children and (perhaps especially) adults to express views that others may view as biased. In addition, by sampling children across a broad, 5‐year age range, we were able to investigate the dynamics of the relationship between parents' and children's gender‐brilliance stereotype—that is, whether this relationship becomes weaker or stronger as children progress through elementary school.

## The intersection of the gender‐brilliance stereotype with race/ethnicity

Gender stereotypes do not apply equally across racial/ethnic groups (Ghavami & Peplau, 2013; Muradoglu et al., 2021; Purdie‐Vaughns & Eibach, 2008). In line with this claim, children's gender‐brilliance stereotypes seem to depend on the racial/ethnic identity of the stereotype targets. For example, Jaxon, Lei, et al. (2019) measured 5‐ and 6‐year‐old U.S. American children's gender‐brilliance stereotypes toward Black and White targets. When asked to select the “really, really smart” person from pairs of women and men from the same racial/ethnic group, 6‐year‐old children exhibited a gender‐brilliance stereotype favoring men only when the targets were White. Children's gender‐brilliance stereotype was reversed when the stimuli depicted Black women and men, such that Black women were chosen more often than Black men as being “really, really smart.” Other evidence, however, suggests that the gender‐brilliance stereotype applies equally across Black and White racial/ethnic groups. Storage et al. (2020) assessed adults' gender‐brilliance stereotype toward Black and White targets using an implicit measure, the IAT. In this study, adults associated both Black and White men with brilliance (and both Black and White women with a range of comparison traits, such as creative and funny) to a comparable degree.

Thus, evidence assessing whether the gender‐brilliance stereotype intersects with race/ethnicity is both sparse and contradictory. In addition, the contradictions are not easy to reconcile: Because the studies by Jaxon, Lei, et al. (2019) and Storage et al. (2020) differed in multiple respects, it is unclear why one found evidence of intersectionality (Jaxon, Lei, et al., 2019) and the other did not (Storage et al., 2020). Is it the age of the participants (children vs. adults, respectively) that explains the discrepancy? Is it the differences in the measures? Clearly, additional evidence is needed to understand the ways in which the gender‐brilliance stereotype intersects with the race/ethnicity of the individuals being stereotyped.

In the current study, which was conducted in Singapore, we examined the question of intersectionality by using an IAT to assess children's and adults' gender‐brilliance stereotypes about both Chinese women and men, who are the majority ethnic group in Singapore (Singapore Department of Statistics, 2021), and White women and men. White individuals make up a small minority of Singapore's population (less than 1%) but nevertheless occupy a high‐status position in Singaporean society, with many White expatriates from European and North American countries employed in high‐ranking, lucrative positions (e.g., Chua et al., 2016; Hof, 2021).

Prior research on intersectional stereotyping motivates competing predictions about whether the gender‐brilliance stereotype will differ across Chinese and White stereotype targets in our Singaporean sample. On the one hand, evidence from the United States suggests that a society's gender stereotypes about the majority racial/ethnic group differ from its gender stereotypes about minority groups (e.g., Biernat & Sesko, 2013; Ghavami & Peplau, 2013; Jaxon, Lei, et al., 2019). From this perspective, we might likewise expect differences in the extent to which Singaporeans associate brilliance with Chinese men (vs. women) and White men (vs. women). On the other hand, there also reasons to expect *similar* levels of gender‐brilliance stereotyping favoring men with respect to these two racial/ethnic groups. One reason is that at least one prior study found evidence of a gender‐brilliance stereotype across different racial/ethnic groups (Storage et al., 2020). In addition, a more theoretically grounded reason for expecting similar levels of gender‐brilliance stereotyping across the two racial/ethnic groups that are our focus here (Chinese and White) is that they are both high‐status groups in Singapore, and high‐status groups are generally stereotyped as being competent (e.g., Fiske et al., 2002). Given that stereotypes about a racial/ethnic group generally apply to the men of that group more than the women (e.g., Ghavami & Peplau, 2013; Purdie‐Vaughns & Eibach, 2008), then perhaps Singaporeans will be equally likely to associate brilliance, a competence‐related trait, with Chinese and White men (vs. women). (To clarify, brilliance—that is, exceptional intellectual ability—is a facet of competence. Even so, these two constructs seem to function differently. For instance, while societal perceptions of women's competence—broadly conceived—have shifted toward equality over time in the United States, Eagly et al., 2020, brilliance is still a stereotypically male attribute.)

Notably, by using a *single measure* to assess gender‐brilliance stereotypes among *both children and adults*, the present research will avoid the interpretive ambiguities that arise when comparing studies that differ simultaneously in the age of the sample and the type of measure used and thus make a meaningful contribution to our understanding of how the gender‐brilliance stereotype intersects with race/ethnicity.

## The gender‐brilliance stereotype in a new national/cultural context

As already described, prior research on the gender‐brilliance stereotype was conducted almost exclusively with participants from the United States, illustrating an unfortunate reality in developmental psychology more generally (Nielsen et al., 2017). The only exception so far is Study 5 from Storage et al. (2020), who assessed gender‐brilliance stereotypes with an IAT among a sample of 514 adults recruited from 78 countries (other than the United States) through the online crowdsourcing platform Mechanical Turk. Although this study found widespread evidence for a gender‐brilliance stereotype, its samples were unlikely to be representative of any of the nations from which participants were recruited, both because the nation‐level samples were small and because the probability of selection bias was high: Participants had to be familiar with a U.S. crowdsourcing platform and know English well enough to pass the language proficiency screener that Storage and colleagues used. In addition, we note that Storage and colleagues recruited only adults; no previous work has examined the gender‐brilliance stereotype among children outside the United States.

In the present research, we examined whether children and adults in Singapore show a gender‐brilliance stereotype. Certain aspects of the cultural context of Singapore suggest it may provide a conservative test of whether the gender‐brilliance stereotype extends beyond the United States. First, the Confucian emphasis on *effort* as the path to success (e.g., Wu, 2006) may mean that brilliance is less culturally salient in Singapore and thus that stereotypes about brilliance are less common. Second, the Confucian emphasis on *fitting in* with others (e.g., Markus & Kitayama, 1991) might also be at odds with gender‐brilliance stereotypes, insofar as thinking that someone is brilliant or a genius (whether they are a man or a woman) implies that they stand out from their peers. However, we also note that the educational system in Singapore tracks students starting as early as elementary school based on their scores on high‐stakes standardized tests (e.g., Hairon, 2022). This element of Singaporean schooling might highlight differences between individuals in their presumed intellectual abilities (in addition to their efforts), which could in turn encourage the formation of brilliance stereotypes. Yet, the most important standardized test for elementary school children in Singapore, the Primary School Leaving Examination, shows an advantage in favor of *girls* (Teng, 2017). Thus, if Singaporean 8‐ to 12‐year‐olds and adults show a gender‐brilliance stereotype favoring males, that would provide strong evidence that this stereotype is cross‐culturally robust.

Finally, we note that, rather than relying on convenience samples recruited online, the present research involved researchers who were intimately familiar with the culture and customs of Singapore to recruit large samples of adults and children through a variety of means (e.g., advertisements, childcare centers, science museums). This strategy ensured that the samples were drawn from across the country and encompassed a relatively diverse cross section of Singaporean society.

## The present research

To summarize, the goals of the present research were to explore three key aspects of the gender‐brilliance stereotype: (1) its trajectory across the elementary school years, (2) the relation between children's and their parents' gender‐brilliance stereotypes, and (3) this stereotype's intersection with stereotypes about race/ethnicity. We report two exploratory studies that addressed these goals in samples of children and adults from Singapore, a context that broadens the geographical and cultural scope of research on the gender‐brilliance stereotype. Studies 1a and 1b served both a methodological purpose, which was to adapt an existing implicit measure of gender‐brilliance stereotypes to the cultural context of Singapore, and a substantive purpose, which was to investigate for the first time whether Singaporean adults exhibit a gender‐brilliance stereotype. Study 2 examined Singaporean elementary school students and their parents and was designed to inform all three of our research goals.

## STUDY 1a

In Study 1a, we adapted the gender‐brilliance IAT created by Storage et al. (2020) to make it suitable for administration in Singapore and then used this IAT to investigate whether Singaporean adults, like U.S. adults, associate brilliance with men more than women. We created two versions of this measure, each administered to approximately half of our sample: a version in which the gender stimuli consisted of photographs of Chinese women and men and another in which the gender stimuli consisted of photographs of White women and men. To provide a broad test of the gender‐brilliance stereotype among Singaporean adults, in Study 1a we recruited both college students and adults from the broader community (specifically, the parents of the children in Study 2).

### Method

#### Participants

A total of 332 Chinese Singaporean adults participated (216 women). The sample consisted of a group of college students (*N* = 201, 129 women, *M*_age_ = 21.29 years, *SD* = 1.84) and a group of parents of elementary school children (*N* = 131, 87 women, *M*_age_ = 42.37 years, *SD* = 4.51). A sensitivity analysis (Faul et al., 2007) indicated that this sample size was sufficient to detect a small effect (Cohen's *d* = 0.15) with 80% power (*α* = .05, two‐tailed test) on a one‐sample *t* test, which is typically used to determine whether *D* scores are significantly above or below the neutral point of 0 in an IAT (Cvencek et al., 2021).

The undergraduate students were recruited via the participant pool at the Nanyang Technological University. Parents were recruited either through online advertisements or at the local science center. Data collection for the present research took place between 2017 and 2018. An additional six participants completed the study but met the IAT exclusion criterion recommended by Greenwald et al. (2003; i.e., had over 10% of responses faster than 300 ms in the IAT), so they were excluded from further analyses. Participants were compensated with either one course credit (college students) or 20 Singapore dollars (SGD; parents) for their time. For this and all subsequent studies, the instructions and stimuli appeared in English, the de facto official language of Singapore. The study was approved by the Institutional Review Board at the Nanyang Technological University.

#### The gender‐brilliance stereotype IAT

To assess the gender‐brilliance stereotype, we used a stereotype IAT—a response time‐based measure of mental associations between groups and traits (e.g., Greenwald et al., 2003). The IAT can identify stereotypes that participants are unaware of or are unwilling to report when asked directly and, as a result, has been used extensively to study stereotypes about sensitive topics (e.g., Nosek et al., 2007). Moreover, the evidence to date suggests that the IAT exhibits strong construct and predictive validity as a measure of stereotypes (see Kurdi et al., 2019).

**FIGURE 1:**
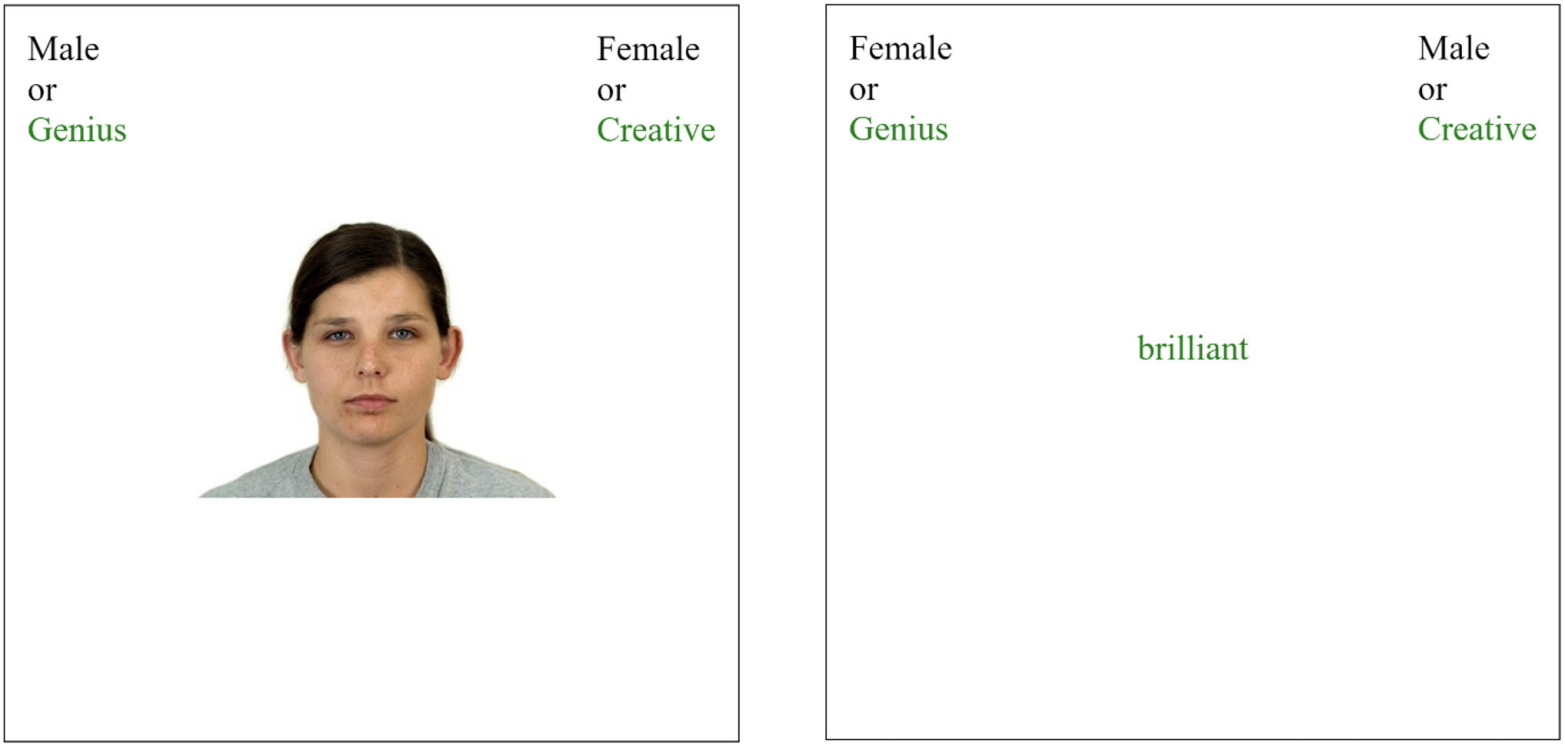
Sample test trials from the gender‐brilliance Implicit Association Test with *creative* as a control attribute and with photographs of White women and men as gender stimuli (Studies 1a and 2). The trial on the left is stereotype‐*congruent*, and the participant is being asked to sort a gender stimulus; the trial on the right is stereotype‐*incongruent*, and the participant is being asked to sort an attribute stimulus. Participants pressed the “E” key to sort a stimulus into the disjunctive category on the left and the “I” key to sort a stimulus into the disjunctive category on the right

Here, we adapted the gender‐brilliance stereotype IAT that was created and validated by Storage et al. (2020) in a U.S. context. Participants were asked to press a key as quickly as possible in order to categorize four types of stimuli: (1) photographs of women's faces, (2) photographs of men's faces, (3) a set of words related to brilliance (i.e., the words “genius,” “brilliant,” and “super‐smart”), and (4) a set of words related to a control attribute (see below). For approximately half of the participants, the individuals depicted in the stimuli were Chinese; for the other half, they were White. Participants sorted the four types of stimuli into two categories, which changed midway through the test: On the *stereotype‐congruent* blocks of trials (see Figure 1), the photographs of men and the words related to brilliance were categorized together (i.e., they were assigned the same response key—say, “E”), and the photographs of women and words related to the control attribute were categorized together (i.e., they were assigned the same response key—say, “I”). On the *stereotype‐incongruent* blocks of trials (see Figure 1), the pairings were reversed (photographs of men + words related to the control attribute vs. photographs of women + words related to brilliance). The order of the stereotype‐congruent and ‐incongruent blocks was counterbalanced across participants (for additional details, see Appendix S1).

If participants associate brilliance with men more than women, they should be faster (and make fewer mistakes) when categorizing stimuli on the stereotype‐congruent trials because the stereotype would make the pairing of men + brilliance more cognitively fluent, facilitating categorization. The difference in average response times between the stereotype‐incongruent and stereotype‐congruent trials, divided by a measure of the variability in response times across the session, is known as a *D* score (for details of the scoring, see Greenwald et al., 2003). For the present IAT, a positive *D* score indicates that the participant associates brilliance with men and the control attribute with women, and a negative *D* score indicates the opposite associations.

A participant's *D* score is a function of both how much they associate brilliance with men *and* how much they associate the control attribute with women. Because our primary interest is in the brilliance–gender association, it is important to ensure that participants' responses on this IAT are not driven primarily by their associations between the control attribute and women. Following Storage et al.'s (2020) recommendation, we used *creative* as a control attribute. This attribute provides a good conceptual match to brilliance (because it is also a desirable intellectual trait), and empirically, the gender‐brilliance IAT with this control attribute yielded a *D* score close to the average of the six IATs in Storage and colleagues' studies (which used six different control attributes), suggesting it may provide a good approximation of the “true” strength of this stereotype. As an additional approach to testing whether the gender‐brilliance IAT captured participants' “brilliance = men” associations per se, we also used the quadruple‐process (quad) model (e.g., Conrey et al., 2005) to disentangle the “brilliance = men” and the “control attribute = women” associations in the IATs used in Studies 1a, 1b, and 2 (for details and results, see Appendix S2).

*D* scores were computed for each participant using the *IATScore* package in R (Storage, 2017), which implements Greenwald et al.'s (2003) scoring algorithm. To assess the reliability of the IAT, we computed two separate *D* scores for each participant—one for the odd trials and the other for the even trials—using the *IATanalytics* package in R (Storage, 2018). The internal consistency statistic calculated using these scores was satisfactory (Cronbach's *α* = .70), suggesting the present IAT is a reliable measure of the gender‐brilliance stereotype.

#### Other measures

Our focus in these studies is on the implicit measure of gender‐brilliance stereotyping, which we also administered to children in Study 2 so that we could examine the age trajectory of this stereotype and the relation between children's and parents' stereotypes on this topic. However, we also administered a set of explicit measures at the end of the sessions in Studies 1a and 1b. These measures are described fully in Appendix S3.

#### Open data and analytic syntax

The raw data and analytic syntax for all studies can be found on the Open Science Framework: https://osf.io/6x9zg/?view_only=8ec93d3e88ff484b8020dceabaa4d612.

### Results

IAT *D* scores were submitted to a linear regression with participant gender (man = 0 vs. woman = 1), participant group (students = 0 vs. parents = 1), target race/ethnicity (White = 0 vs. Chinese = 1), and all possible interactions as predictors. These variables were mean‐centered to facilitate the interpretation of the lower‐order coefficients. IAT block order (incongruent‐first = 0 vs. congruent‐first = 1) was entered as a covariate because this variable typically accounts for some of the variability in IAT scores (e.g., Greenwald et al., 2003).

**FIGURE 2:**
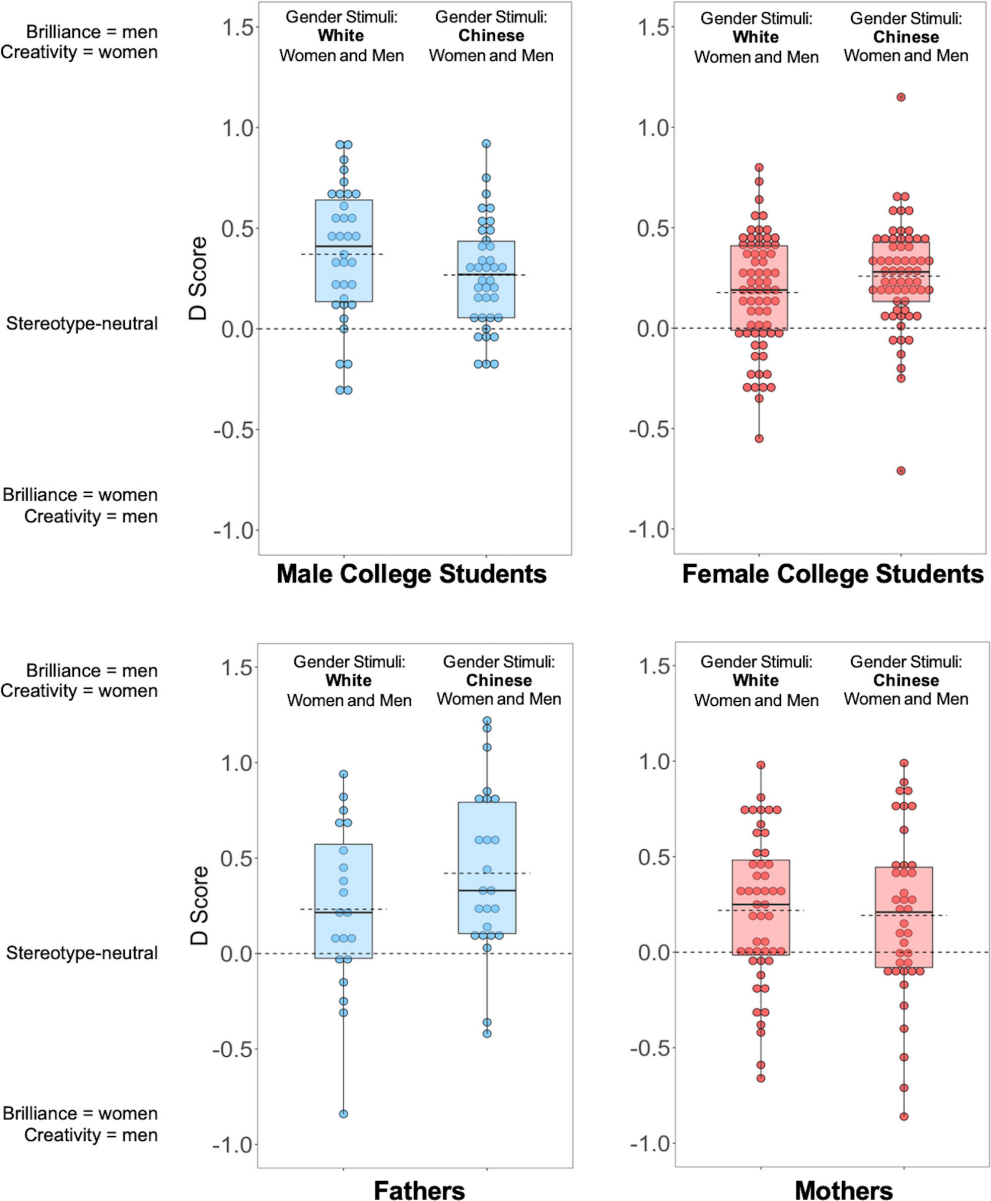
College students' (top) and parents' (bottom) Implicit Association Test *D* scores in Study 1a. Lower and upper ends of the boxes represent the 25% and 75% percentiles; dots represent individual participants' scores; dashed lines are the means; solid lines are the medians

**FIGURE 3:**
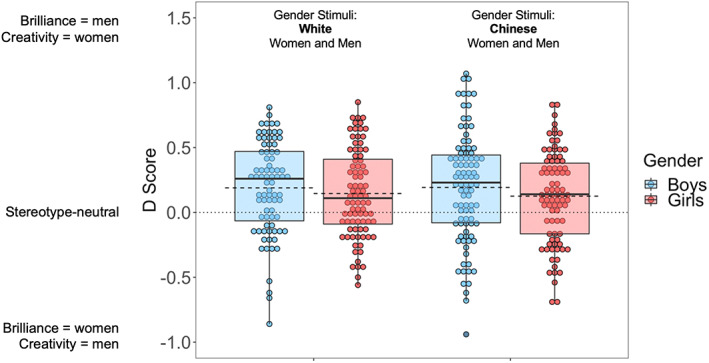
Children's Implicit Association Test *D* scores in Study 2. Lower and upper ends of the boxes represent the 25% and 75% percentiles; dots represent individual children's scores; dashed lines are the means; solid lines are the medians

The linear regression was computed with the *regress* command in Stata 14.1 (StataCorp, 2015), specifying heteroskedasticity‐robust standard errors. Based on the output of the regression, we computed a range of follow‐up tests (sometimes referred to as “marginal tests”) using Stata's *margins* command. In this and all subsequent studies, we report marginal (or adjusted) means, calculated with *margins* as well, rather than observed means (but see Figures 2 and 3 for observed means and medians). All marginal means are accompanied by 95% confidence intervals (CIs). In line with other work using the IAT, we also report Cohen's *d*s for the marginal tests of the mean *D* values against 0. These *d* statistics were computed by dividing the *t* values derived from marginal tests by √*N*, where *N* is the size of the full sample or the relevant subsample (e.g., women, students; see Lakens, 2013). All *t* values reported here and in all subsequent studies have *N* − *k* − 1 degrees of freedom, where *N* is the size of the full sample in the relevant regression model and *k* is the number of parameters being estimated. In Study 1a, all *t* values had 332 − 8 − 1 = 323 degrees of freedom.

To test whether Singaporean adults showed a gender‐brilliance stereotype, we compared their *D* scores against zero. The mean *D* score was .25 [.22, .29], which was significantly above zero, *t* = 13.72, *p* < .001, *d* = 0.75, indicating a gender‐brilliance stereotype favoring men (see Figure 2). Notably, this *D* score is almost identical to that reported by Storage et al. (2020) in their U.S. sample, *M* = .24 [.21, .27], *d* = 0.72, and comparable in magnitude to the *D* scores from IATs measuring gender‐science and gender‐career stereotypes (e.g., *d*s = 0.93 and 1.10, respectively, in Nosek et al., 2007).

Both women's *D* scores, *M* = .21 [.17, .26], *t* = 9.87, *p* < .001, *d* = 0.67, and men's *D* scores, *M* = .32 [.25, .39], *t* = 9.62, *p* < .001, *d* = 0.89, were significantly above zero. Men's gender‐brilliance stereotype was stronger than women's, *B* = −.11 [−.18, −.03], *p* = .008. In addition, both students' *D* scores, *M* = .25 [.22, .29], *t* = 12.79, *p* < .001, *d* = 0.90, and parents' *D* scores, *M* = .25 [.18, .32], *t* = 7.05, *p* < .001, *d* = 0.62, were significantly above zero, and nearly identical, *B* = −.01 [−.09, .07], *p* = .84.

The perceived race/ethnicity of the women and men in the photographs did not affect *D* scores, *B* = .03 [−.04, .10], *p* = .43. Participants showed a gender‐brilliance stereotype both when the photographs were of Chinese individuals, *M* = .27 [.22, .32], *t* = 10.49, *p* < .001, *d* = 0.82, and when the photographs were of White individuals, *M* = .24 [.19, .29], *t* = 8.98, *p* < .001, *d* = 0.69 (see Figure 2).

We also observed an unexpected three‐way interaction between participant gender (women vs. men), participant group (students vs. parents), and target race/ethnicity (White vs. Chinese), *B* = −.43 [−.77, −.08], *p* = .015. Among male participants, the two‐way participant group × target race/ethnicity interaction was significant, *B* = .30 [.01, .59], *p* = .046, with students showing stronger gender‐brilliance stereotypes when the photographs depicted White individuals but parents showing stronger gender‐brilliance stereotypes when the photographs depicted Chinese individuals (see Figure 2). The three‐way interaction emerged because this two‐way interaction was not present among female participants, *B* = −.13 [−.32, .06], *p* = .17. However, these differences were not predicted and are difficult to interpret, so we will not discuss them further.

Finally, the regression revealed the typical effect of IAT block order, *B* = .21 [.13, .28], *p* < .001. Participants who saw the stereotype‐congruent blocks first (*M* = .35 [.30, .40], *t* = 14.03, *p* < .001, *d* = 1.09) had higher *D* scores than participants who saw the stereotype‐incongruent blocks first (*M* = .15 [.10, .20], *t* = 5.62, *p* < .001, *d* = 0.44). However, the *D* scores were significantly above zero for both groups, *p*s < .001. This order effect is common in IAT research (e.g., Greenwald et al., 2003) and is thought to arise from the cognitive inertia that accompanies the switch in sorting rules midway through the test. For instance, if participants start with the stereotype‐congruent blocks, they will be slower on the subsequent stereotype‐incongruent blocks not just because the pairing of the categories in these blocks is incongruent with their stereotypic associations (e.g., *female* and *genius*) but also because the sorting rules have changed. This will magnify the response time differences between the stereotype‐incongruent and ‐congruent blocks and inflate *D* scores. Through the same mechanisms, the opposite block order typically deflates *D* scores.

### Discussion

Using an IAT adapted from one previously used primarily on U.S. samples (Storage et al., 2020), we found that Singaporean adults show a robust gender‐brilliance stereotype that favors Chinese and White men (vs. women), which indicates that the gender‐brilliance stereotype, previously investigated almost exclusively in the United States, is also present in at least one other culture (Singapore) that differs from the United States in relevant respects (e.g., its Confucian emphasis on effort as the key to success and on fitting in with others rather than standing out).

In addition, the results of Study 1a suggest that the “brilliance = men” association applies to a similar extent to members of two different racial/ethnic groups, even though one of the groups is a small minority in Singaporean society (White) and the other is the overwhelming majority (Chinese). This finding aligns with that of Storage et al. (2020), who observed a robust gender‐brilliance stereotype with gender stimuli from both minority and majority racial/ethnic groups in the United States. The present result is also consistent with theoretical arguments that high‐status groups—and, in particular, the men of those groups—are positively stereotyped with respect to competence‐related traits (Fiske et al., 2002; Ghavami & Peplau, 2013).

## STUDY 1b

Because of the relative nature of the IAT, the positive *D* scores observed in Study 1a could in principle be due to an association between *creative* (the control attribute) and women. To explore this possibility, in Study 1b we administered a version of the gender‐brilliance stereotype IAT in which the control attribute was switched to *happy*, which is suitable both because it is likely gender‐neutral (e.g., Bem, 1974; Helmreich et al., 1981; but see Hess et al., 2004) and because it matches *genius* in desirability (see Appendix S1). If a gender‐brilliance stereotype (i.e., a positive *D* score) is observed with this control attribute as well, that will bolster confidence in the conclusion that we are capturing the “brilliance = men” association per se.

### Method

#### Participants

We recruited 57 Chinese Singaporean undergraduates (39 women, *M*_age_ = 21.30 years, *SD* = 1.27) from the participant pool at the Nanyang Technological University. One additional participant was excluded because they went faster than 300 ms for more than 10% of the trials during the IAT (Greenwald et al., 2003). A sensitivity analysis indicated that this sample size was sufficient to detect a small‐to‐medium effect (Cohen's *d* = 0.38) with 80% power (*α* = .05, two‐tailed test) on a one‐sample *t* test.

#### Procedure and materials

The procedure and materials were identical to those used in Study 1a, except that we switched the control attribute to *happy* (stimuli: “happy,” “joyful,” and “super‐upbeat”) and we only administered the IAT with photographs of Chinese women and men as gender stimuli. This version of the IAT demonstrated satisfactory reliability (Cronbach's *α* = .70, as in Study 1a).

### Results

IAT *D* scores were submitted to a linear regression with participant gender (man = 0 vs. woman = 1) as a substantive predictor and block order (incongruent‐first = 0 vs. congruent‐first = 1) as a covariate. All *t* tests reported in this study had 54 degrees of freedom.

Overall, *D* scores on this gender‐brilliance IAT with *happy* as a control attribute were significantly above zero, *M* = .24 [.17, .31], *t* = 6.59, *p* < .001, *d* = 0.87. It is also noteworthy that these scores were similar to those obtained in Study 1a with *creative* as a control attribute, *M* = .25 [.22, .29], *d* = 0.75, and to those obtained by Storage et al. (2020) in the United States with *happy* as a control attribute, *M* = .19 [.16, .22], *d* = 0.59.

Both women, *M* = .27 [.18, .37], *t* = 6.03, *p* < .001, *d* = 0.97, and men, *M* = .17 [.05, .29], *t* = 2.76, *p* = .008, *d* = 0.65, showed a gender‐brilliance stereotype, and there was no significant gender difference in this study, *B* = .11 [−.04, .26], *p* = .16. The effect of block order was not significant in this study, *B* = .12 [−.02, .27], *p* = .094, but the means went in the same direction as in Study 1a (when the stereotype‐incongruent blocks were first: *M* = .18 [.06, .29], *t* = 3.16, *p* = .003, *d* = 0.60; when the stereotype‐congruent blocks were first: *M* = .30 [.21, .40], *t* = 6.39, *p* < .001, *d* = 1.19).

### Discussion

Study 1b provided evidence for the construct validity of the gender‐brilliance IAT as a measure of “brilliance = men” stereotypic associations: The *D* scores across Studies 1a and 1b were similar despite the change in control attribute from *creative* to *happy*. Thus, the present results also reinforce the conclusion that Singaporean adults implicitly believe men are more likely than women to possess an exceptional level of intellectual ability.

## STUDY 2

Study 2 investigated the gender‐brilliance stereotype favoring Chinese and White men (vs. women) in a large sample of Singaporean children aged 8 to 12 and their parents.

### Method

#### Participants

We recruited 342 Chinese Singaporean children (171 girls) aged 8 to 12 years (*M*_age_ = 10.11 years, *SD* = 1.29, range = 8.00–12.94 years) from childcare centers, science museums, and through online advertisements. Of the 342 children, 168 were tested together with their parents (*n* = 131; 68% mothers; same sample as in Study 1a). Ninety‐six parents had one child in our sample; 33 parents had two children in our sample; and two parents had three children in our sample. The sample was socioeconomically diverse, with the most commonly reported monthly household income brackets being 5000–9999 SGD (37%), 10,000–19,999 SGD (25%), and 2500–4999 SGD (20%).

A sensitivity analysis indicated that this sample size (*N* = 342) had 80% power (*α* = .05, two‐tailed test) to detect a small effect (Cohen's *d* = 0.15) on a one‐sample *t* test, which can establish whether children show a gender‐brilliance stereotype, and a small‐to‐medium‐sized relationship between children's stereotypes and age (*r* = .15). In addition, the sample of parent–child dyads (*N* = 168) had 80% power (*α* = .05, two‐tailed test) to detect a small‐to‐medium‐sized relationship between parents' and children's stereotypes (*r* = .21).

An additional seven children's data were excluded because they had difficulties understanding the words or following the rules of the IAT (*n* = 2), because of experimenter error (*n* = 2), or because of a server error that prevented the data from being saved (*n* = 3). Past studies that have used the IAT with child samples have also excluded participants based on error rates, using thresholds of 30% (e.g., Galdi et al., 2014) or 35% (e.g., Cvencek et al., 2011). In the current study, four children had greater than 35% error rates in the test blocks. To be conservative, we retained their data in our analyses. We note that excluding these participants did not alter our conclusions (see Appendix S4).

Parental consent and child assent were obtained prior to the study for all child participants. Informed consent was obtained from all parents who participated.

#### Procedure

Children were tested individually using an Asus Chrome Notebook PC (Model C302C). The session took place in either a separate room or a quiet corner at the testing venue (e.g., childcare center, science center, library). Each session began with a 3‐ to 5‐min training, during which the experimenter ensured that the child was able to properly read and define the six attribute stimuli in the IAT. The words were presented to children in a randomized order. After this training, children were told that they would be playing a sorting game (i.e., the IAT). The experimenter went through the IAT instructions with the child and checked that the child understood them. The experimenter then administered the IAT. The sessions were conducted in English and were video‐recorded. After the IAT, children were also asked an exploratory open‐ended question, “What do you want to be when you grow up?” Because a sizable number of children said they did not know or reported unconventional career aspirations (e.g., YouTuber, roller‐coaster tester), we will not consider responses to this question further here. At the end of the sessions, children were thanked and given a small gift (e.g., a pen).

#### The gender‐brilliance stereotype IAT

We administered the gender‐brilliance IAT with *creative* as the control attribute. The IAT was identical to the one used in Study 1a. Children completed either the IAT with photographs of Chinese individuals (*n* = 178) or the IAT with photographs of White individuals (*n* = 164). Parents always completed the same version of the IAT as their children to allow for direct comparison. The internal consistency of the IAT was satisfactory for both children and their parents (Cronbach's *α*s = .72 and .79, respectively).

### Results and discussion

#### Singaporean children's gender‐brilliance stereotype: Age trajectory and intersectionality

Children's *D* scores were submitted to a linear regression with participant gender (boy = 0 vs. girl = 1), participant age (in years with two‐decimal precision), target race/ethnicity (White = 0 vs. Chinese = 1), and all possible interactions as predictors. As before, IAT block order (incongruent‐first = 0 vs. congruent‐first = 1) was entered as a covariate. All *t* tests reported in this section had 333 degrees of freedom.

The average *D* score for the entire sample was .16 [.13, .20], which was significantly above zero, *t* = 9.20, *p* < .001, *d* = 0.50, indicating that Singaporean children—like Singaporean adults—associate brilliance with men more than women (see Figure 3). Both girls' *D* scores, *M* = .13 [.09, .17], *t* = 5.73, *p* < .001, *d* = 0.44, and boys' *D* scores, *M* = .19 [.14, .25], *t* = 7.21, *p* < .001, *d* = 0.55, were significantly above zero. The difference between girls' and boys' gender‐brilliance stereotypes did not reach statistical significance, *B* = −.06 [−.13, .004], *p* = .067, and neither did any of the interactions between participant gender and the other variables, *p*s > .74. We observed again the typical effect of IAT block order, in the same direction as for the adults in Studies 1a and 1b, *B* = .36 [.29, .43], *p* < .001.

One of our goals here was to investigate the age trajectory of the gender‐brilliance stereotype. Relevant to this goal, we found that the strength of the gender‐brilliance stereotype increased with age in our sample, *B* = .04 [.01, .07], *p* = .006 (see Figure S2). With each additional year of age, the average *D* score increased by .04 points (equivalent to 0.10 *SD*s). None of the interactions of age with the other variables were significant, *p*s > .57.

Given that we had access to Singaporean adults' responses on the same IAT (Study 1a), we also asked whether the oldest children in our sample displayed the gender‐brilliance stereotype at levels already comparable to those of adults or, alternatively, whether additional increases in the strength of this stereotype were likely to occur before children reached adulthood. To answer this question, we used the regression model above to predict children's gender‐brilliance stereotype at the top of the age range in our sample—namely, at age 13. (Our oldest child participant was 12.94 years old.) The predicted *D* score at this age was .27 [.19, .36], which is almost identical to adults' average *D* score in Study 1a, where we also administered the gender‐brilliance IAT with *creative* as the control attribute, *M* = .25 [.22, .29]. Thus, it seems likely that only minimal changes occur in the strength of children's gender‐brilliance stereotype between age 13 and adulthood.

Another goal of the present research was to investigate whether the magnitude of the gender‐brilliance stereotype varies as a function of the stereotype targets' racial/ethnic groups. *D* scores were significantly above zero both when the gender stimuli in the IAT consisted of Chinese women and men, *M* = .16 [.11, .21], *t* = 6.17, *p* < .001, *d* = 0.46, and when they consisted of White women and men, *M* = .16 [.12, .21], *t* = 6.98, *p* < .001, *d* = 0.54. The gender‐brilliance stereotype did not differ between the two target race/ethnicities, *B* = −.003 [−.07, .07], *p* = .94, and none of the interactions involving this variable were significant either, *p*s > .57.

#### Singaporean children's gender‐brilliance stereotype: Relation to parents' stereotype

To investigate the relation between children's and parents' gender‐brilliance stereotypes, we used the data from the subset of 168 children who participated with their parents. Children's *D* scores were submitted to a linear regression with their parents' *D* scores, child gender (boy = 0 vs. girl = 1), child age (in years with two‐decimal precision), and all possible interactions as predictors. This model included three covariates as well: the block order for the child's IAT (incongruent‐first = 0 vs. congruent‐first = 1), the block order for the parent's IAT (incongruent‐first = 0 vs. congruent‐first = 1), and target race/ethnicity (White = 0 vs. Chinese = 1). In an alternative specification of this analysis, target race/ethnicity was included in the factorial part of the model, fully interacted with the other three predictors. In this model, none of the terms involving target race/ethnicity were significant. Thus, we included this variable as a covariate instead. The other results remain materially the same whether the interaction terms between target race/ethnicity and the other variables are included or omitted.

To account for the potential dependencies among the *D* scores from siblings, the regression models reported in this section used cluster–robust standard errors (StataCorp, 2015), which relax the assumption of independence of the observations. However, the results were almost identical to those obtained when this adjustment was not made. (Because information about which participants were siblings was only available for the children who were tested together with their parents, we were unable to make this adjustment to the standard errors in the preceding analyses with the full sample of children.)

This model revealed a significant positive relationship between parents' and children's *D* scores, *B* = .12 [.02, .23], *p* = .021. To illustrate the magnitude of this relationship, a 1 *SD* increase in parents' *D* scores corresponded to a 0.16 *SD* increase in children's *D* scores.

**FIGURE 4:**
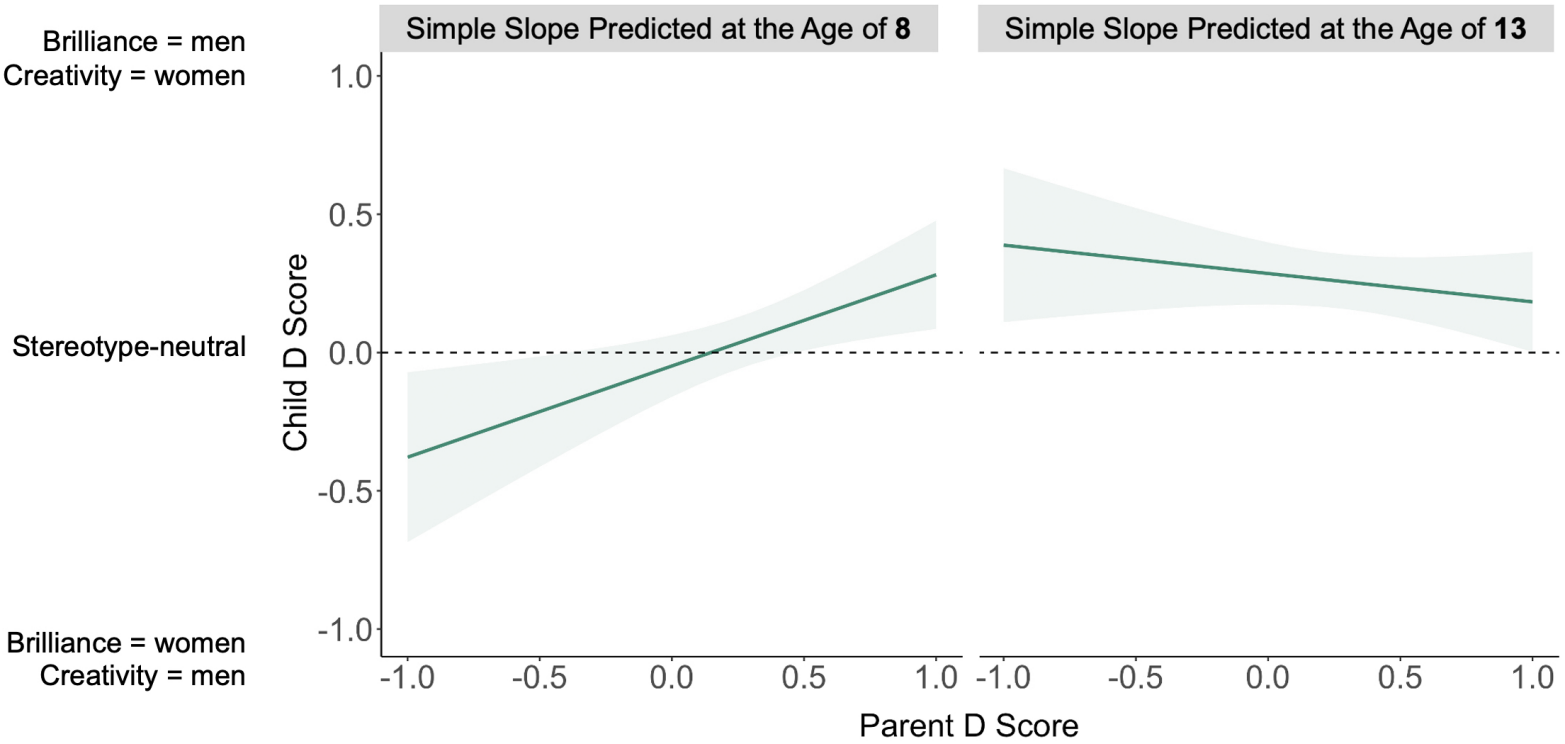
The relationship between parents' and children's Implicit Association Test *D* scores, predicted at the bottom (left panel) and top (right panel) of the age range in our study (that is, ages 8 and 13, respectively). At the age of 8, children's gender‐brilliance stereotypes increased as a function of their parents' stereotypes. At the age of 13, children's gender‐brilliance stereotypes were unrelated to parents' stereotypes. Shaded areas represent 95% CIs

We also observed a significant interaction between parents' stereotypes and children's age, which suggested that the relationship between parents' and children's *D* scores was stronger for younger than for older children, *B* = −.09 [−.15, −.02], *p* = .008. To unpack this interaction, we used the *margins* command in Stata to calculate the predicted relationship between parents' and children's stereotypes at the bottom and top of the age range in our study (that is, at ages 8 and 13, respectively; see Figure 4). Parents' stereotypes were a significant predictor of children's stereotypes at age 8, *B* = .33 [.14, .52], *p* = .001. At this age, a 1 *SD* increase in parents' *D* scores corresponded to a 0.42 *SD* increase in children's *D* scores. In contrast, this relationship was not significant at age 13, *B* = −.10 [−.29, .09], *p* = .28.

Finally, the regression model revealed a three‐way interaction between parents' stereotypes, children's age, and children's gender, *B* = .14 [.01, .28], *p* = .040 (see Figure S3). To unpack this three‐way interaction, we examined the two‐way, parent stereotype × child age interaction among girls and boys separately. This interaction was significant for boys, *B* = −.16 [−.25, −.08], *p* < .001, for whom the positive relationship between their stereotypes and their parents' stereotypes declined significantly with age, but not for girls, *B* = −.01 [−.10, .09], *p* = .90, for whom the positive relationship between their stereotypes and their parents' stereotypes did not decline with age.

In two additional regression analyses (described fully in Appendix S5), we explored whether the association between parents' and children's gender‐brilliance stereotypes was moderated by parents' gender (68% of children participated in the study with their mothers; 32% with their fathers) and primary caregiver status (65% of children participated in the study with a primary caregiver; 35% with a secondary caregiver). We found no evidence of moderation by parent gender (for similar results, see Degner & Dalege, 2013; Tenenbaum & Leaper, 2002). However, children's (in particular, girls') gender‐brilliance stereotypes were more closely related to their parents' stereotypes when the parents with whom they participated in the study were their primary caregivers. Arguably, primary caregivers spend more time with children and thus have more opportunities to convey their beliefs to them.

## GENERAL DISCUSSION

The present research investigated the stereotype that associates brilliance and genius with men more than women. Although this stereotype presents an obstacle to women's advancement in many prestigious careers (e.g., Bian, Leslie, & Cimpian, 2018; Bian, Leslie, Murphy, et al., 2018; Meyer et al., 2015; Muradoglu et al., 2021), many aspects of it remain poorly understood. Our findings contribute to scientific knowledge of this stereotype in three key respects: They provide evidence on how the strength of the gender‐brilliance stereotype changes with age; they identify a potential source of this stereotype in children's environments (namely, their parents); and they speak to whether this stereotype applies in similar versus different ways across racial/ethnic groups. We summarize and discuss each of these contributions in turn, also highlighting some of the limitations of the present evidence and the directions for future work that it suggests.

Previous research on the gender‐brilliance stereotype suggested that this stereotype emerges at around the age of 6 (at least in the United States; Bian, Leslie, & Cimpian, 2018; Bian et al., 2017) but left open the question of whether and how this stereotype changes over the course of childhood. Our data are the first to identify that this stereotype increases in strength over the course of the elementary school years until reaching adult levels around the age of 13. Of note, the claim that the gender‐brilliance stereotype reaches adult levels at age 13 is based on a statistical extrapolation—we did not recruit adolescent participants older than 12. Given that other gender stereotypes continue to change over adolescence (e.g., Liben & Bigler, 2002; Miller et al., 2018), it will be important for future research to investigate this period of development directly. Another important caveat is that, at this point, we do not know whether our findings regarding the age trajectory of the gender‐brilliance stereotype are specific to the cultural context of Singapore or whether similar age trajectories would be identified elsewhere as well. Pursuing this question is also a worthwhile goal for future work.

It is noteworthy that this increase in the strength of the gender‐brilliance stereotype over the elementary school years was identified with an implicit measure of stereotyping—namely, an IAT. This measure has several characteristics that made it well suited for our purposes here. For example, the IAT can be administered in the exact same form to both adults and children above a certain age, whereas many other measures—especially those reliant on eliciting agreement or disagreement with various statements—often need to be adapted when administered to different age groups (e.g., Degner & Dalege, 2013). In addition, the fact that the IAT can reveal whether individuals associate a certain group with a certain trait without needing to ask them to self‐report on this association is a major asset to any investigation of sensitive topics such as gender stereotypes.

However, the IAT has drawbacks as well. In particular, our use of the “standard” IAT, which relies on four distinct categories of stimuli (typically, two social groups and two attributes), meant that we had to include a control attribute in addition to brilliance. As a result, the IAT estimated participants' association of brilliance with men *relative to* their association of another attribute with women. Alternative IAT formats exist that do not have this feature—most notably, the single‐category IAT (SC‐IAT). This version of the IAT uses only three categories of stimuli; in our case, these would have been the two gender categories and brilliance‐related words. However, the validity of the SC‐IAT has been questioned (e.g., Kurdi et al., 2019) because participants can use shortcuts when completing it, meaning that their responses do not always map onto their gender–trait associations: For instance, if participants are sorting *male* and *genius* together with one key and *female* separately with another key, they can choose to categorize the stimuli simply based on whether they match *female*, which would lead their response times to be independent of whether they associate *genius* and *male*. Thus, we believe that the disadvantages of using the SC‐IAT outweigh its potential advantages as a simpler measure of stereotypic associations. We also note that the present (standard) gender‐brilliance IAT revealed virtually the same *D* scores with two different, and relatively gender‐neutral, control attributes (*creative* and *happy*), suggesting that participants' responses on this test were likely driven by their associations between brilliance and men (for additional validation data, see Storage et al., 2020). Finally, this conclusion was reinforced by the results of the quad models, which revealed evidence for a “brilliance = men” association that was independent of the “control attribute = women” association (see Appendix S2).

The second major contribution of this work is to identify a relation between parents' and children's gender‐brilliance stereotypes, which implicates parents as one of the potential sources of children's stereotypes on this topic. Parents are the primary socialization agents of their children (e.g., Maccoby, 1992; Tenenbaum & Leaper, 2002). Although parents may be somewhat unlikely to express explicit gender stereotypes in front of their children, given that overt prejudice has become less socially acceptable over the last few decades (e.g., Swim et al., 1995), they may still convey beliefs about gender differences in intellectual abilities indirectly through subtle aspects of their language and parenting practices. For example, they may praise girls for being hardworking and boys for being smart when children excel in school (e.g., Yee & Eccles, 1988); they may express superficially egalitarian views that nevertheless covertly signal that boys are more capable (e.g., “Girls are just as smart as boys”; Chestnut et al., 2021); or they may spend more time discussing intellectually challenging topics, such as math and science, with their sons than with their daughters (e.g., Chang et al., 2011; Crowley et al., 2001). In future work, it would be fruitful to dig deeper and understand which aspects of parent–child interactions reveal parents' gender‐brilliance stereotypes to their children and what factors prompt children to adopt these views or reject them.

One meaningful moderator in this respect seems to be children's age: We found a reliable relation between parents' and children's gender‐brilliance stereotypes among the younger children in our sample, but this relation waned as children approached adolescence (especially among boys). Adolescence marks a time of increasing psychological distance, and sometimes even conflict, between children and their parents (e.g., Qu et al., 2016). It seems likely that these developmental processes would lead to a decrease in the extent to which children's views on gender are aligned with those of their parents. However, this finding appears to contrast with the conclusion of a recent meta‐analysis of studies on parents' and children's intergroup attitudes, which found stronger correlations with parental attitudes among older, not younger, children (Degner & Dalege, 2013). What explains this discrepancy? An important caveat about Degner and Dalege's finding is that it was confounded by the similarity between the measures administered to parents and those administered to younger versus older children: The older were the children, the more similar were the measures they completed to the measures that their parents completed, which explains why the correlations were stronger among this age group. When this confound was removed, children's age was no longer a significant moderator of the relationship between parents' and children's attitudes. Thus, our finding of a tighter relationship between parents' and children's stereotypes among younger children is not as unexpected as one might initially assume based on the results of Degner and Dalege's meta‐analysis.

Another noteworthy moderator of the relation between children's and parents' gender‐brilliance stereotypes was whether the participating parent was the child's primary caregiver: Parents' and children's gender‐brilliance stereotypes were more closely related among the subset of children (specifically, girls) who participated with their primary caregivers. This finding addresses a question that is often asked about socialization research: Do the observed correlations between parents' and children's attitudes on a topic arise because of the influence of parents' socialization practices on their children or instead because parents and children are genetically related, which may predispose them to think in similar ways (e.g., Harris, 2011)? The finding that parents' primary caregiver status moderates the relation between their and children's gender‐brilliance stereotypes argues against a genetic relatedness‐based explanation for the present findings—parents' genetic relatedness to their children does not differ as a function of their status as primary versus secondary caregivers. Of course, it is also possible that the relation between children's and parents' stereotypes is due to an effect of *children's* beliefs on their parents. However, such child‐to‐parent effects seem less plausible in light of the fact that, in the present research, the relation between children's and parents' gender‐brilliance stereotypes was stronger among *younger* children. Arguably, children's beliefs should influence their parents' when children are old enough to articulate these beliefs in a way that might persuade their parents, yet we found the opposite.

All this being said, it is also important to acknowledge that the cross‐sectional nature of our design precludes strong conclusions about parental stereotypes having a causal influence on children's stereotypes. It may be, for example, that parents and children mutually reinforce each other's stereotypes or that the correlation between their stereotypes is due to a third factor (e.g., the degree of exposure to relevant cultural messages). Since experimental designs on this topic (i.e., manipulating parents' stereotypes to assess their effects on children) are unethical, we look forward to future work that uses longitudinal designs and measures a broader range of inputs. Such research would be able to determine whether parents' stereotypes uniquely predict increases in their children's stereotypes over time, above and beyond other relevant factors.

Our focus here has been on parents' stereotypes, but children's stereotypes may of course be shaped by other sources as well (e.g., media, peers, teachers), and the influence of these sources (relative to parents) is likely to increase with age, potentially explaining the observed increase in the strength of children's gender‐brilliance associations. For instance, teachers also view boys as more intellectually gifted than girls (e.g., Avitzour et al., 2020; J. Cimpian et al., 2016), a notion that is reinforced by messages in children's broader cultural environments, from the text on McDonald's Happy Meal boxes (Hourigan, 2020) to the dialogue in children's movies (Gálvez et al., 2019). Research examining whether exposure to these messages predicts (increases in) children's gender‐brilliance stereotypes would be informative.

The third contribution of the present work is to provide new evidence on whether the association between brilliance and men applies across racial/ethnic groups. In general, the content of gender stereotypes differs as a function of the other social identities that a person is perceived to embody (e.g., race/ethnicity, sexuality; e.g., Ghavami & Peplau, 2013; Purdie‐Vaughns & Eibach, 2008). However, the gender‐brilliance stereotype—at least when measured implicitly—seems to apply broadly: We observed similar levels of this stereotype with respect to Chinese and White men (vs. women) in the present research, and Storage et al. (2020) found that the gender‐brilliance stereotype favors Black men (vs. women) as well, at least in the United States.

While it is of course possible that future work will reveal more variability in how this stereotype applies across racial/ethnic groups, another possibility is that the very structure of the IAT makes intersectionality effects harder to observe. In the gender‐brilliance IAT, for example, participants' job is to sort as fast as possible photographs of women and men *by gender*—nothing else about these individuals' perceived identities is relevant from a participant's perspective. This aspect of the IAT may prompt participants to ignore other beliefs and assumptions they may have otherwise activated about the individuals in the photographs and focus exclusively on their membership in a gender category. In contrast, in the above‐mentioned study that found intersectional gender‐brilliance stereotypes, Jaxon, Lei, et al. (2019) did not draw explicit attention to the gender of the stereotype targets. In fact, they tried to draw children's attention *away* from gender by including several trials on which participants had to guess who the “really, really smart” person was from a pair of individuals of the same gender (and race/ethnicity). It is perhaps because this task did not minimize the salience of racial/ethnic identity, as the gender‐brilliance IAT arguably did, that Jaxon, Lei, and colleagues found that children's gender‐brilliance stereotypes differentiated between racial/ethnic groups. This possibility is reinforced by research published while the present manuscript was in the final stages of the review process. Using a task similar to that of Jaxon, Lei, et al., Shu et al. (2022) reported that children from mainland China and the United States showed a gender‐brilliance stereotype favoring men when children evaluated White targets but a reverse, “brilliance = women” association for Asian targets, much like Jaxon, Lei, and colleagues found for Black targets.

Notably, this argument is similar to one made recently by Petsko et al. (2022), according to which intersectional stereotypes are more often observed in contexts where multiple identities of the stereotype targets (vs. just one such identity) are accessible to observers and relevant to their goals. For example, Petsko and colleagues found intersectional stereotypes even in an IAT when participants sorted social stimuli using multiple‐identity labels (e.g., “Black women”) rather than single‐identity labels (e.g., “women”). In future work, it would be worthwhile to adapt this strategy for investigating the gender‐brilliance stereotype from an intersectional perspective as well.

Finally, this research also contributes to the growing literature on the gender‐brilliance stereotype by suggesting that this stereotype is present in at least one other national/cultural context beyond the United States. Of course, the fact that this stereotype is observed in two countries does not mean that it is universal. However, the substantial cultural and geopolitical differences between Singapore and the United States make the present studies a particularly informative data point with respect to the cross‐cultural generalizability of the gender‐brilliance stereotype.

## Supporting information


Appendix S1.
