# Supplementary material for: The acquisition of the gender‐brilliance stereotype: Age trajectory, relation to parents' stereotypes, and intersections with race/ethnicity
Source: Child Dev. 2022 May 30;93(5):e581–97. doi: 10.1111/cdev.13809 (PMC9545489; doi:10.1111/cdev.13809)
Supplement: Supplementary file 1 — Appendix S1. [file CDEV-93-e581-s001.pdf]

The Acquisition of the Gender-Brilliance Stereotype: Age Trajectory, Relation to Parents' Stereotypes, and Intersections with Race/Ethnicity

SUPPLEMENTARY ONLINE MATERIALS

Appendix S1: Additional Information about the Gender-Brilliance IAT 2

Appendix S2: Quad Model Estimates for Studies 1a, 1b, and 2 5

Appendix S3: The Explicit Measures in Studies 1a and 1b 7

Figure S2: The Separate Age Trajectories of Girls' and Boys' Gender-Brilliance Stereotypes .. 10

Figure S3: Conditional Effects of Age and Parent Stereotype on Girls' and Boys' Stereotypes 11

Appendix S4: The Results of Study 2 After Excluding Children with High IAT Error Rates 12

Appendix S5: Do Parents' Gender and Primary Caregiver Status Moderate the Relationship between Parents' and Children's Gender-Brilliance Stereotypes? 14

References 16

Appendix S1:

Additional Information about the Gender-Brilliance IAT

Block Structure

The IAT started with two practice blocks (20 trials each). On each trial of these blocks, participants sorted *either* gender stimuli *or* attribute stimuli into the corresponding categories: In one practice block, participants sorted photographs of women and men into the categories *female* and *male*. In the other practice block, participants sorted words related to brilliance and creativity into the categories *genius* and *creative*. On each trial, one stimulus (i.e., photograph or word) appeared in the middle of the screen, and participants had to press the “E” or “I” key to indicate whether the stimulus belonged to the category on the left (“E”) or the one on the right (“I”). To move on to the next stimulus, participants had to press the correct key (i.e., the key that corresponded to the designated category of the stimulus). Otherwise, a red X appeared at the bottom of the screen to indicate that the response was incorrect.

The following two blocks (i.e., the third and the fourth) were test blocks (40 trials each). On each trial of these blocks, participants sorted *both* gender stimuli *and* attribute stimuli into the corresponding disjunctive categories (e.g., *male* or *genius*). The four gender and trait labels that defined the disjunctive categories (*female, male*, *genius,* and *creative*) appeared in pairs in the top left and right corners of the screen (see Figure 1 in the main text). A gender category was always paired with an attribute label; the specific pairings depended on whether the trial occurred in the stereotype-congruent block (*male* or *genius, female* or *creative*) or the stereotype-incongruent block (*female* or *genius*, *male* or *creative*).

In the next (fifth) block, the sides of the gender categories were switched, and participants had an opportunity to practice sorting gender stimuli into the *female* and *male*

categories in their new locations for 20 trials.

The IAT ended with two more test blocks (40 trials each) in which, because of the location change of the gender categories, the gender–attribute pairings were reversed relative to the first set of test blocks.

Stimuli

The gender stimuli consisted of photographs of eight young adult female faces and eight young adult male faces (see Figure 1 in the main text). For approximately half of the participants, the individuals depicted were Chinese; for the other half, they were White. The Chinese individuals were all students at [*blinded for review*] who agreed to have their photograph taken for this study in exchange for 5 SGD. The White individuals were all selected from the Chicago Face Database (Ma et al., 2015). All depicted individuals had neutral facial expressions. The women and men in the photographs were rated as equally attractive by a separate group of Singaporean adults we recruited for this purpose (N = 31; see Figure S1 for details). These participants also rated the *genius* stimuli (the words “genius,” “brilliant,” and “super-smart”) and the *creative* stimuli (the words “creative,” “artistic,” and “super-imaginative”) as equally desirable and positive.

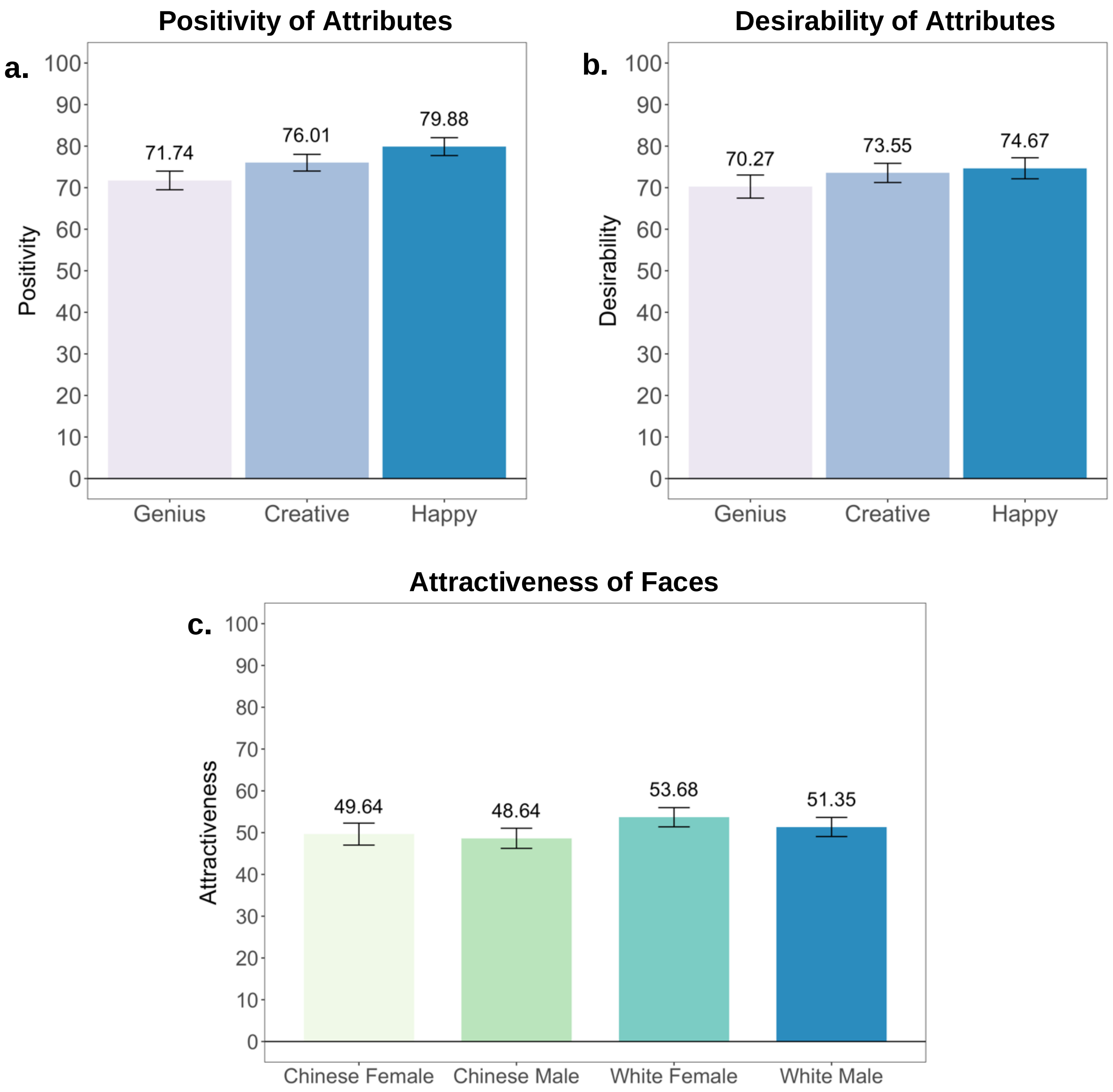

Figure S1. In the norming study, Singaporean adults (N = 31) rated the word and face stimuli that appeared in the IATs using a 100-point slider scale. Higher scores represent greater positivity/desirability of the words and greater attractiveness of the faces. Mean positivity ratings of the attributes are shown in Figure S1a; mean desirability ratings of the attributes are shown in Figure S1b; and mean ratings of attractiveness for the gender stimuli are shown in Figure S1c. Error bars represent standard errors. “Genius” and “creative” words were rated as comparable in terms of positivity, $t(30) = -1.80$, $p = .082$, and desirability, $t(30) = -1.34$, $p = .191$. “Genius” and “happy” words were rated as comparable in terms of desirability, $t(30) = -1.77$, $p = .086$. “Happy” was rated as more positive than “genius,” $t(30) = -3.78$, $p < .001$. Chinese male and female faces were rated as equally attractive, $t(30) = 0.65$, $p = .518$. White male and female faces were also rated as equally attractive, $t(30) = 1.60$, $p = .121$.

Appendix S2:

Quad Model Estimates for Studies 1a, 1b, and 2

Because the associations measured by the standard IAT are always relative, we turned to the quadruple-process (quad) model (Conrey et al., 2005) to disentangle the “brilliance = men” and the “control attribute = women” associations. The quad model is a multinomial processing tree that uses error rates (rather than response times) to compute six probability estimates from the IAT for each sample.[1] Each parameter represents a cognitive process that feeds into the error rates in the IAT. Most important for our purposes are the parameters that index the activation of cognitive associations (i.e., the AC parameters; Conrey et al., 2005). The two AC parameters in the gender-brilliance IAT represent the “brilliance = men” association and the “creativity/happy = women” association, respectively. Using these AC parameters, we tested if the association of interest, “brilliance = men,” was observed. The parameters from the quad model for Studies 1a, 1b, and 2 are displayed in Table S1 below.

In Study 1a, the overall model fit decreased when the “brilliance = men” AC parameter was constrained to be zero, $\chi^2(1) = 206.90$, $p < .001$, suggesting that this parameter independently contributed to the error rates in the IAT. This result thus indicated the presence of a “brilliance = men” association. Additionally, a significant “creativity = female” association was also observed, $\chi^2(1) = 163.77$, $p < .001$. Using the same model-comparison technique, we found significant “brilliance = men” and “control attribute = women” associations in both Studies 1b and 2 (see Table S1). Overall, the findings from the quad model support the construct

[1] Rather than generating estimates for each individual participant, the quad model uses the overall error rate to estimate a set of parameters for *the entire sample*.

validity of the gender-brilliance IAT: This IAT captured the “brilliance = men” association across studies rather than just the “control attribute = women” association.

Table S1

Quad Model Parameter Estimates across Studies

**Process parameter**	**Association**	**Estimate**
Study 1a (Adults)		
AC	Brilliance + men	0.05***
	Creative + women	0.05***
OB		1.00***
D		0.86***
G		0.54***
Study 1b (Adults)		
AC	Brilliance + men	0.04***
	Happy + women	0.06***
OB		1.00***
D		0.83***
G		0.49
Study 2 (Children)		
AC	Brilliance + men	0.04***
	Creative + women	0.03***
OB		1.00***
D		0.77***
G		0.52***

Note. AC = association activation (activation of a stereotypical association); OB = overcoming bias (ability to overcome the biased associations); D = discriminability (ability to accurately differentiate the types of stimuli); G = guessing (right/left key bias when making random guesses). *** $p < .001$.

Appendix S3:

The Explicit Measures in Studies 1a and 1b

We reasoned that explicit measures would be less well suited for our purposes in this research because social desirability concerns would likely suppress overt endorsement of the gender-brilliance stereotype among older children and adults (see Storage et al., 2020). To assess whether this concern is valid, at the end of the sessions in Studies 1a and 1b we administered an explicit measure on this topic (e.g., "One is more likely to find a male with a genius-level IQ than a female with a genius-level IQ"; 1 = *strongly disagree* to 9 = *strongly agree*; Bian et al., 2018b), as well as three scales assessing explicit attitudes toward women: a 20-item Liberal Feminist Attitude and Ideology Scale (LFAIS, Morgan, 1996; Storage et al., 2020), a 5-item Old-fashioned Sexism Scale (Swim et al., 1995), and an 8-item Modern Sexism Scale (Swim et al., 1995). Items were adapted to fit the Singapore context by removing content that would be unfamiliar to Singaporean participants. Participants rated their agreement with each statement on a 6-point scale, which ranged from 1 = *strongly disagree* to 6 = *strongly agree*. Higher scores on the LFAIS represent stronger feminist ideologies. Higher scores on the Old-fashioned Sexism Scale and the Modern Sexism Scale represent stronger sexist beliefs. The means and correlation matrices for Studies 1a and 1b are provided in Table S2 below.

We expected that, due in part to concerns about appearing biased, (1) participants would be hesitant to endorse the gender-brilliance stereotype on the explicit measure, and (2) there would be only a weak relationship between responses on this measure and responses on the gender-brilliance IAT. This prediction was borne out in Studies 1a and 1b, as detailed below.

Study 1a

Endorsement of the gender-brilliance stereotype on the explicit measure was close to the

midpoint of the 1–9 scale (i.e., 5), $M = 5.18$, $SD = 1.50$. To compare participants' gender-brilliance stereotypes when measured explicitly vs. implicitly, we first computed a Cohen's d for the difference between each measure's mean and its respective neutral point (i.e., the midpoint of 5 for the explicit measure and 0 for the IAT). We could then use these *d*s and their 95% *CI*s to compare (approximately) the magnitude of the gender-brilliance stereotype across the two measures. Consistent with our expectation, the explicit measure, $d = 0.12$ [0.01, 0.23], indicated substantially weaker gender-brilliance stereotypes than the implicit measure, $d = 0.77$ [0.66, 0.89].[2] In addition, the two measures were uncorrelated, $r(298) = -.02$, $p = .80$ (see also Table S2).

Study 1b

In Study 1b, endorsement of the gender-brilliance stereotype on the explicit measure was below the midpoint of the 1–9 scale, $M = 4.59$, $SD = 1.39$, indicating *disagreement*. Thus, the effect size for the explicit stereotype measure, $d = -0.29$ [−0.56, −0.03], was in the opposite direction of that for the IAT, $d = 0.85$ [0.59, 1.12], which again revealed substantial levels of the stereotypic "brilliance = men" association. In addition, as in Study 1a, there was no significant correlation between the implicit and explicit measures, $r(55) = -.08$, $p = .58$ (see Table S2). This evidence reinforces the conclusion that the IAT is better suited for measuring a sensitive topic such as the gender-brilliance stereotype among Singaporean adults.

Summary

On the whole, this evidence confirmed our concerns about using an explicit gender-brilliance stereotype measure among adults and validated our decision to use an implicit measure

[2] We performed this comparison on the subset of participants who had valid responses on both measures (excluding the 32 participants who missed the attention checks embedded in the explicit measures; see footnote to Table S2). In addition, the Cohen's *d*s in this Appendix were calculated from the observed D scores (rather than from the t values of marginal tests, as in the main text) because this method made it straightforward to calculate confidence intervals. The Cohen's d values calculated through these two methods were very similar.

for our joint investigation of parents' and children's gender-brilliance stereotypes in Study 2.

Table S2

Means, Standard Deviations, Internal Consistencies, and Bivariate Correlations Between the Implicit and Explicit Measures in Studies 1a and 1b

			**Study 1a**				
Variables	*M* (*SD*)	α	1	2	3	4	5
1. IAT	0.25 (0.35)	.70	—				
2. Endorsement	5.18 (1.50)	.88	–0.02	—			
3. Awareness	5.42 (1.59)	.91	0.06	0.39***	—		
4. Feminism	4.41 (0.51)	.83	–0.05	–0.09	–0.18**	—	
5. Old-fashioned Sexism	2.32 (0.75)	.64	0.05	0.36***	0.27***	–0.42***	—
6. Modern Sexism	3.42 (0.72)	.79	0.04	0.13*	0.26***	–0.65***	0.34***
			**Study 1b**				
Variables	*M* (*SD*)	α	1	2	3	4	5
1. IAT	0.24 (0.28)	.70	—				
2. Endorsement	4.59 (1.39)	.88	–0.08	—			
3. Awareness	5.76 (1.36)	.89	0.12	0.48***	—		
4. Feminism	4.45 (0.49)	.85	0.08	–0.28*	–0.03	—	
5. Old-fashioned Sexism	2.25 (0.72)	.69	–0.04	0.51***	0.28*	–0.28*	—
6. Modern Sexism	3.30 (0.69)	.80	–0.06	0.50***	0.07	–0.61***	0.30*

Note. *N*s = 297–300 in Study 1a and 57 in Study 1b. Two attention check questions were included among the items in the explicit measures to identify inattentive participants. Participants who missed both checks (32 in Study 1a, 0 in Study 1b) were excluded from analyses of the explicit responses. * $p < .05$; ** $p < .01$; *** $p < .001$.

Figure S2:

The Separate Age Trajectories of Girls' and Boys' Gender-Brilliance Stereotypes

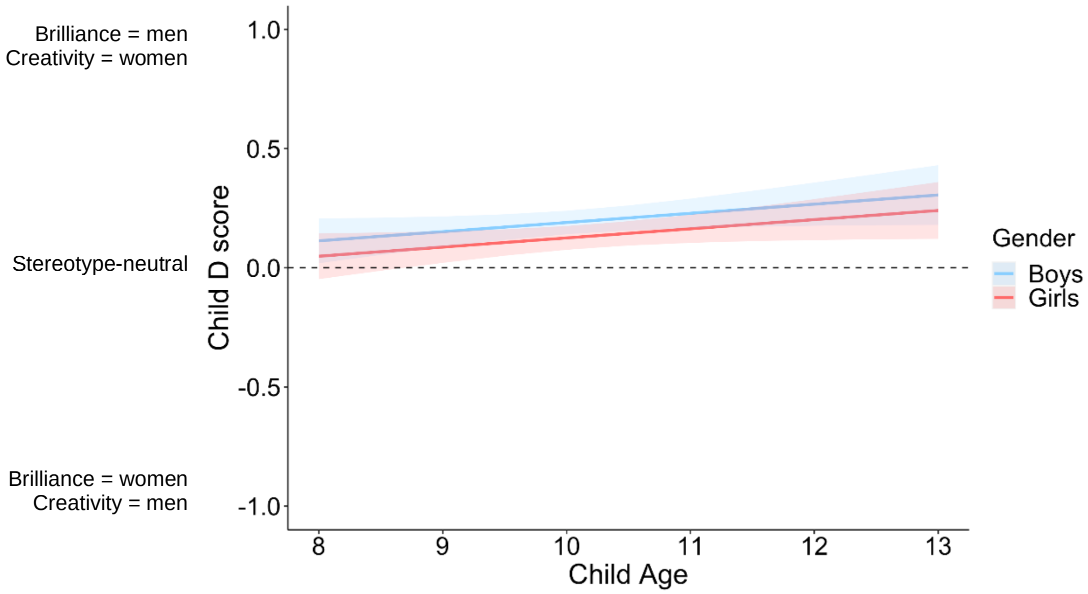

Figure S2. Girls' and boys' *D* scores as a function of age, controlling for target race/ethnicity (Chinese vs. White) and block order. Shaded areas represent 95% *CI*.

Figure S3:

Conditional Effects of Age and Parent Stereotype on Girls' and Boys' Stereotypes

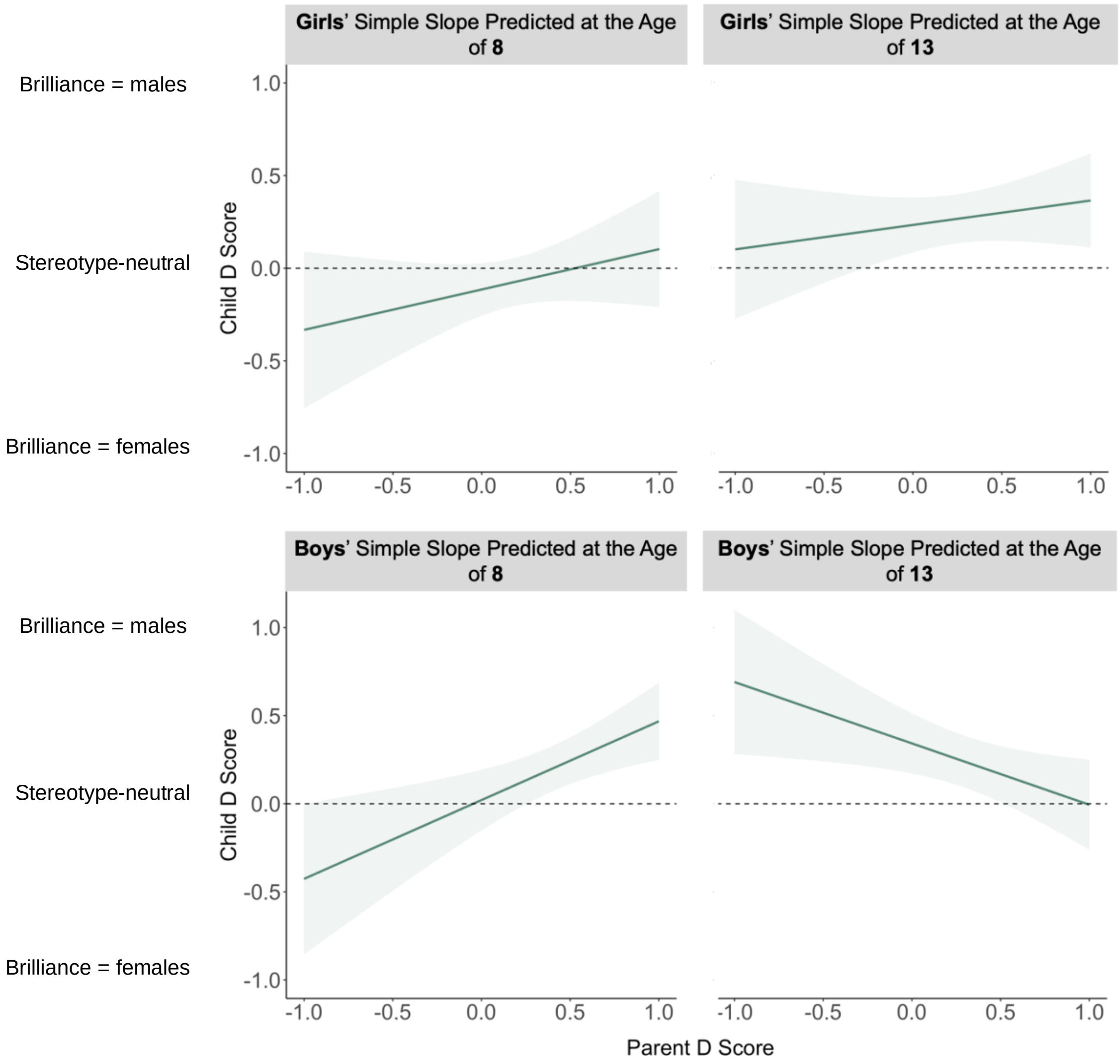

Figure S3. Predicted conditional effects of age and parent IAT *D* score on girls' and boys' IAT *D* scores, controlling for target race/ethnicity and IAT block order. Girls' (top panel) *D* scores increased as a function of their parents' *D* scores, irrespective of age. The association between boys' (bottom panel) and their parents' *D* scores waned with age.

Appendix S4:

The Results of Study 2 After Excluding Children with High IAT Error Rates

One possible concern about administering IATs to children is that children may not be able to correctly categorize the face and word stimuli, which may undermine the reliability and validity of their IAT scores. Past studies have addressed this concern by excluding child participants based on their IAT error rates (e.g., Cvencek et al., 2011; Galdi et al., 2014). In Study 2, four child participants had an error rate higher than 35% in the test blocks. After excluding these four participants, the sample size of Study 2 became 338 (169 girls, M_{age} = 10.10 years, *SD* = 1.28, *range* = 8.00–12.94). Below, we report results of Study 2 after excluding these four participants.

Age Trajectory and Intersectionality

Similar to the results reported in the main text, both girls, *M* = 0.13 [0.09, 0.18], *t* = 5.91, *p* < .001, *d* = 0.45, and boys, *M* = 0.20 [0.14, 0.25], *t* = 7.14, *p* < .001, *d* = 0.55, had IAT scores above the neutral point of zero. Children's *D* scores increased as a function of age, *B* = 0.04 [0.01, 0.06], *p* = .011, with a one year increase in age corresponding to a 0.10 *SD* increase in children's *D* scores. Children's *D* scores did not differ based on the race/ethnicity of the targets, *B* = 0.002 [−0.07, 0.07], *p* = .963: Children showed a gender-brilliance stereotype both when the gender stimuli were Chinese women and men, *M* = 0.17 [0.11, 0.22], *t* = 6.23, *p* < .001, *d* = 0.47, and when the gender stimuli were White women and men, *M* = 0.16 [0.12, 0.21], *t* = 7.00, *p* < .001, *d* = 0.55.

Relation to Parents' Stereotype

We again found a significant positive relationship between parents' and children's *D* scores, $B = 0.12$ [0.01, 0.23], $p = .026$, with a 1 *SD* increase in parents' *D* scores corresponding to a 0.16 *SD* increase in children's *D* scores. The association between children's and parents' *D* scores was moderated by child age, $B = –0.08$ [–0.15, –0.02], $p = .013$. Parents' *D* scores predicted children's *D* scores at age 8, $B = 0.32$ [0.12, 0.51], $p = .002$, but did not predict children's scores at age 13, $B = –0.10$ [–0.29, 0.10], $p = .33$.

In short, removing children who had higher than 35% error rate in the IAT did not change the results of Study 2.

Appendix S5:

Do Parents' Gender and Primary Caregiver Status Moderate the Relationship between Parents' and Children's Gender-Brilliance Stereotypes?

Parents' Gender. We explored whether parents' gender moderates the association between parents' and children's gender-brilliance stereotypes. Previous research has suggested that mothers' and fathers' gender attitudes and stereotypes may relate differentially to children's own stereotypes (Croft et al., 2014; del Río et al., 2018; but see Degner & Dalege, 2013; Tenenbaum & Leaper, 2002). Of the parents in our study, 68% were mothers and 32% were fathers. Parent gender (man = 0 vs. woman = 1) was added to the model described in the main text, fully interacted with parents' *D* scores, child gender, and child age. The model revealed no significant interaction terms involving parent gender, *p*s > .19, indicating that parent-child stereotype association did not differ by parent gender (see OSF for full regression results).

Parents' Primary Caregiver Status. We explored whether parents' status as a primary caregiver moderates the association between parents' and children's gender-brilliance stereotypes. Of the parents in our study, 65% were their participating children's primary caregivers and 35% were not. Primary caregiver status was determined with one item: "Who are the major caretakers of your child?" Participants could check all that apply from among the following options: mother, father, grandparents, maid,[3] and others.

Similar to the model described above, parent caregiver status (non-caregiver = 0 vs. caregiver = 1) was added to the regression model, interacting with parents' *D* scores, child gender, and child age. The model revealed a three-way interaction between parents' caregiver

[3] Live-in domestic workers ("maids"), often from lower-income countries from Southeast Asia, are common in Singapore, especially among dual-career couples. According to a recent estimate (Awang & Wong, 2019), approximately 20% of all households in Singapore employ a maid.

status, parents' gender-brilliance stereotypes, and children's gender, $B = 0.56$ [0.20, 0.91], $p = .002$. To unpack this three-way interaction, we examined the two-way interaction between parents' caregiver status and their stereotypes separately for girls and boys. This two-way interaction was significant only among girls, $B = 0.31$ [0.02, 0.59], $p = .034$ (among boys: $B = -0.23$ [−0.52, 0.06], $p = .13$): Girls' gender-brilliance stereotypes were positively related to parents' stereotypes when their parents were the primary caregiver, $B = 0.26$ [0.11, 0.42], $p = .001$. In contrast, no association was observed between girls' and parents' gender-brilliance stereotypes when the parents with whom they participated in the study were not their primary caregivers, $B = -0.04$ [−0.31, 0.22], $p = .74$ (see OSF for full regression results).

References

Awang, N., & Wong, P. T. (2019, November 4). The Big Read: As maids become a necessity for many families, festering societal issues could come to the fore. *Channel News Asia*. Retrieved from: https://www.channelnewsasia.com/singapore/maids-foreign-domestic-workers-singapore-necessity-families-847201

Conrey, F. R., Sherman, J. W., Gawronski, B., Hugenberg, K., & Groom, C. J. (2005). Separating multiple processes in implicit social cognition: The quad model of implicit task performance. *Journal of Personality and Social Psychology*, *89*(4), 469–487. https://doi.org/10.1037/0022-3514.89.4.469

Cvencek, D., Meltzoff, A. N., & Greenwald, A. G. (2011). Math-gender stereotypes in elementary school children. *Child Development*, *82*(3), 766–779. https://doi.org/10.1111/j.1467-8624.2010.01529.x

Galdi, S., Cadinu, M., & Tomasetto, C. (2014). The roots of stereotype threat: When automatic associations disrupt girls' math performance. *Child Development*, *85*(1), 250–263. https://doi.org/10.1111/cdev.12128

Ma, D. S., Correll, J., & Wittenbrink, B. (2015). The Chicago face database: A free stimulus set of faces and norming data. *Behavior research methods*, *47*(4), 1122–1135. https://doi.org/10.3758/s13428-014-0532-5

Morgan, B. L. (1996). Putting the feminism into feminism scales: Introduction of a Liberal Feminist Attitude and Ideology Scale. *Sex Roles*, *34*(5-6), 359–390. https://doi.org/10.1007/BF01547807

Storage, D., Charlesworth, T. E. S., Banaji, M. R., & Cimpian, A. (2020). Adults and children implicitly associate brilliance with men more than women. *Journal of Experimental Social Psychology*, *90*, 104020. https://doi.org/10.1016/j.jesp.2020.104020

Swim, J. K., Aikin, K. J., Hall, W. S., & Hunter, B. A. (1995). Sexism and racism: Old-fashioned and modern prejudices. *Journal of Personality and Social Psychology, 68*(2), 199–214. https://doi.org/10.1037/0022-3514.68.2.199
